# The role of intraoperative tranexamic acid in transurethral resection of the prostate: a systematic review and meta-analysis of randomised-controlled trials

**DOI:** 10.1007/s11255-026-05180-7

**Published:** 2026-05-07

**Authors:** Cian M. Hehir, Gavin G. Calpin, Gavin P. Dowling, Gordon R. Daly, Barry B. McGuire

**Affiliations:** 1 Department of Surgery, Royal College of Surgeons, 123 St. Stephen’s Green, Dublin, Ireland; 2 Department of Urology, St. Vincents University Hospital, Elm Park, Dublin 4, Ireland

**Keywords:** Tranexamic acid, Transurethral resection of prostate, Benign prostatic hyperplasia, Surgical complications, Blood loss, Operative time, Blood transfusion

## Abstract

**Purpose:**

To critically appraise and evaluate the safety and efficacy of intraoperative tranexamic acid (TXA) administration during transurethral resection of the prostate (TURP).

**Methods:**

A systematic search of online databases was conducted to identify randomised-controlled trials (RCTs) which compared surgical outcomes and complication rates in patients undergoing TURP for benign prostatic hyperplasia (BPH) who were treated with TXA (intervention) as compared to placebo/none (control). The efficacy of intraoperative TXA was evaluated through outcomes related to blood loss, rate of blood transfusion and operative time. The safety of TXA was evaluated through pooled analysis of both deep venous thrombosis and pulmonary emboli.

**Results:**

Nine RCTs met the inclusion criteria for this meta-analysis in which a total of 661 patients underwent TURP for BPH (331 TXA: 330 Control). There was significantly less intraoperative bleeding in the TXA group (MD –40.23 mL [95%CI –66.76 to –13.71], *p* = 0.003), which was reflected in a significantly lesser haemoglobin drop on the first postoperative day (MD –0.55 g/dL [95%CI –0.71 to –0.39], *p* < 0.00001). TXA was associated with a significantly shorter operative time (MD –9.77 min [95%CI –16.97 to –2.58], *p* = 0.008), with patients who received TXA exposed to a significantly reduced risk of blood transfusion (0.99% TXA vs. 7.69% Control, OR 0.16 [95%CI 0.03–0.93], *p* = 0.04). There was no statistically significant increase in risk of DVT in the TXA group, *p* = 0.46.

**Conclusion:**

Intraoperative administration of TXA is safe and effective in reducing intraoperative blood loss, operative time and postoperative haemoglobin (Hb) drop with resultant decrease in blood transfusion requirements. This meta-analysis did not detect any significant increase in venous thrombosis or risk of pulmonary embolism incurred by TXA administration.

**Supplementary Information:**

The online version contains supplementary material available at https://doi.org/10.1007/s11255-026-05180-7.

## Introduction

Benign prostatic hyperplasia (BPH) refers to a non-malignant enlargement of prostatic smooth muscle and epithelial cells, occurring predominantly within the transitional zone and periurethral areas of the prostate gland [1]. The enlargement of periurethral glandular tissue presents commonly with lower urinary tract symptoms (LUTS), and contributes to a significant global disease burden, with 30% of men above 50 years of age suffering from moderate to severe LUTS secondary to BPH [2]. The lifetime global prevalence of BPH is reported at 26.2% and results in significant associated disability as demonstrated by the mean disability adjusted life years (DALYs) of 22.36 years [3, 4]. Although much of the clinical burden of BPH can be managed successfully with conservative measures and pharmacological therapy, surgical intervention will be required in one in five men with BPH [5].

Despite the introduction of numerous novel minimally invasive techniques, TURP remains the reference standard in surgical management of BPH [6]. Even with the significant evolution of operative technologies relevant to TURP in the past decades, bleeding remains a common perioperative complication, especially in the context of an enlarged, congested prostate gland [7]. The advent of bipolar TURP and improved sophistication of endoscopic apparatus has significantly reduced perioperative bleeding and blood transfusion rates [8, 9]. However, intraprocedural blood loss during TURP remains at 2.4–4.6 mL/min [10]. Intraoperative bleeding during TURP can impede endoscopic visualisation, prolong operative time and result in increased patient morbidity [11, 12]. Similarly, bleeding in the postoperative period may result in prolonged bladder irrigation, resultant delays in decatheterisation and discharge, and possibly, albeit uncommon, a return to the operating theatre. As such, limiting blood loss in the context of TURP presents potential to simultaneously improve both patient outcomes and institutional productivity, and is of increased interest in urological literature [13].

Tranexamic acid (TXA) is an antifibrinolytic agent which has shown promising capacity to limit perioperative blood loss across a broad range of surgical interventions [14, 15]. Widespread perioperative use of TXA, however, has been limited by concern surrounding potential for increased risk of thrombotic events. Large scale randomised-controlled trials (RCTs), such as the landmark POISE-3 trial, have demonstrated a 25% reduction in major bleeding events without significant increase in thrombotic complications [16]. A growing body of evidence has been published evaluating the role of intraoperative TXA in TURP. This study aims to critically evaluate the existing evidence base to assess the safety and clinical efficacy of intraoperative TXA in TURP.

## Methods

This systematic review and meta-analysis was performed in accordance with the Preferred Reporting Items for Systematic Reviews and Meta-Analyses (PRISMA) guidelines [17]. All authors contributed to the formation of the study protocol, which was prospectively registered with the Prospective Register of Systematic Reviews (PROSPERO—CRD42024611563).

### Population, Intervention, Control, Outcome (PICO) framework

*Population*. Adult, male patients undergoing TURP for BPH.

*Intervention*. Intraoperative administration of tranexamic acid during TURP for BPH.

*Control*. Placebo or no TXA administered during TURP for BPH.

*Outcome.* Primary outcomes include operative time, endoscopic quality of view, intraoperative blood loss, postoperative blood loss, incidence of blood transfusion, and incidence of thrombotic events. Secondary outcomes include time to discontinuation of continuous bladder irrigation, time to decatheterisation, and time to discharge.

### Search strategy and outcome

A systematic search of online electronic databases (PubMed Medline, Scopus & Embase) was performed by two independent reviewers (CMH and GGC). The search terms: (tranexamic acid), (transurethral resection) and (prostate) were combined using the Boolean operator ‘AND’. The search was not limited by year of publication. Duplicate studies were manually removed (CMH) prior to screening of titles, abstracts and full texts. Relevant papers for inclusion were identified using a predetermined search strategy devised by senior author (BBM). Studies were screened for inclusion by two independent reviewers (CMH and GGC) with disagreements resolved through consensus by a third reviewer (BBM). Retrieved studies were reviewed to ensure studies met inclusion criteria for a minimum of one outcome of interest. The reference lists of the included studies were manually screened to identify additional relevant papers for inclusion. The final search was completed on October 5th, 2025.

#### Inclusion criteria

1. Randomised-controlled trials comparing patients undergoing TURP treated with TXA to patients treated with placebo/no TXA.
2. TXA given in the intraoperative period.
3. At least one outcome of interest is reported in both intervention and control groups.
4. Full text available for review in English or French language.

#### Exclusion criteria

1. Non-randomised-controlled trial study design.
2. TXA given in the preoperative or postoperative period only, without intraoperative administration.
3. Inadequate statistical information provided for inclusion in meta-analysis.
4. Full text not available.
5. Published abstracts from conference proceedings, review articles, case reports and editorial articles.

### Data extraction

The following data were extracted and collated from studies meeting the inclusion criteria: (1) First author name, (2) year of publication, (3) journal of publication, (4) study design, (5) country of origin, (6) number of patients – intervention/control/total, (7) outcomes of interest expressed as mean + /– standard deviations (SD) for continuous variables, and (8) outcomes of interest expressed as incidence for dichotomous variables. Data were extracted in duplicate by two independent reviewers (CMH and GGC) with consensus sought from a third reviewer (BBM) in cases of disagreement.

### Statistical analysis

Data were expressed as dichotomous or binary outcomes, reported as odds ratios (ORs) or mean differences (MDs) expressed with 95% confidence intervals (CIs) following estimation using the Mantel–Haenszel method. Statistical heterogeneity was determined using *I*^2^ statistics. Either fixed or random effects models were applied based on whether significant heterogeneity (*I*^2^ > 50%) existed between studies included in the analysis. All tests of significance were two-tailed with *p* < 0.05 indicating statistical significance. Meta-analysis was performed using Review Manager (RevMan), Version 5.4 (Nordic Cochrane Centre, Copenhagen, Denmark) [18]. Risk-of-bias assessment was performed in accordance with the Cochrane guidelines through utilisation of risk of bias for randomised trials (RoB2) assessing the included studies for the presence of bias across five key domains: bias arising from the randomisation process, bias from deviations from intended interventions, bias due to missing outcome data, bias in measurement of outcome, and bias in selection of the reported results. Representative figures generated using *robvis* visualisation software.

## Results

**Fig. 1:**
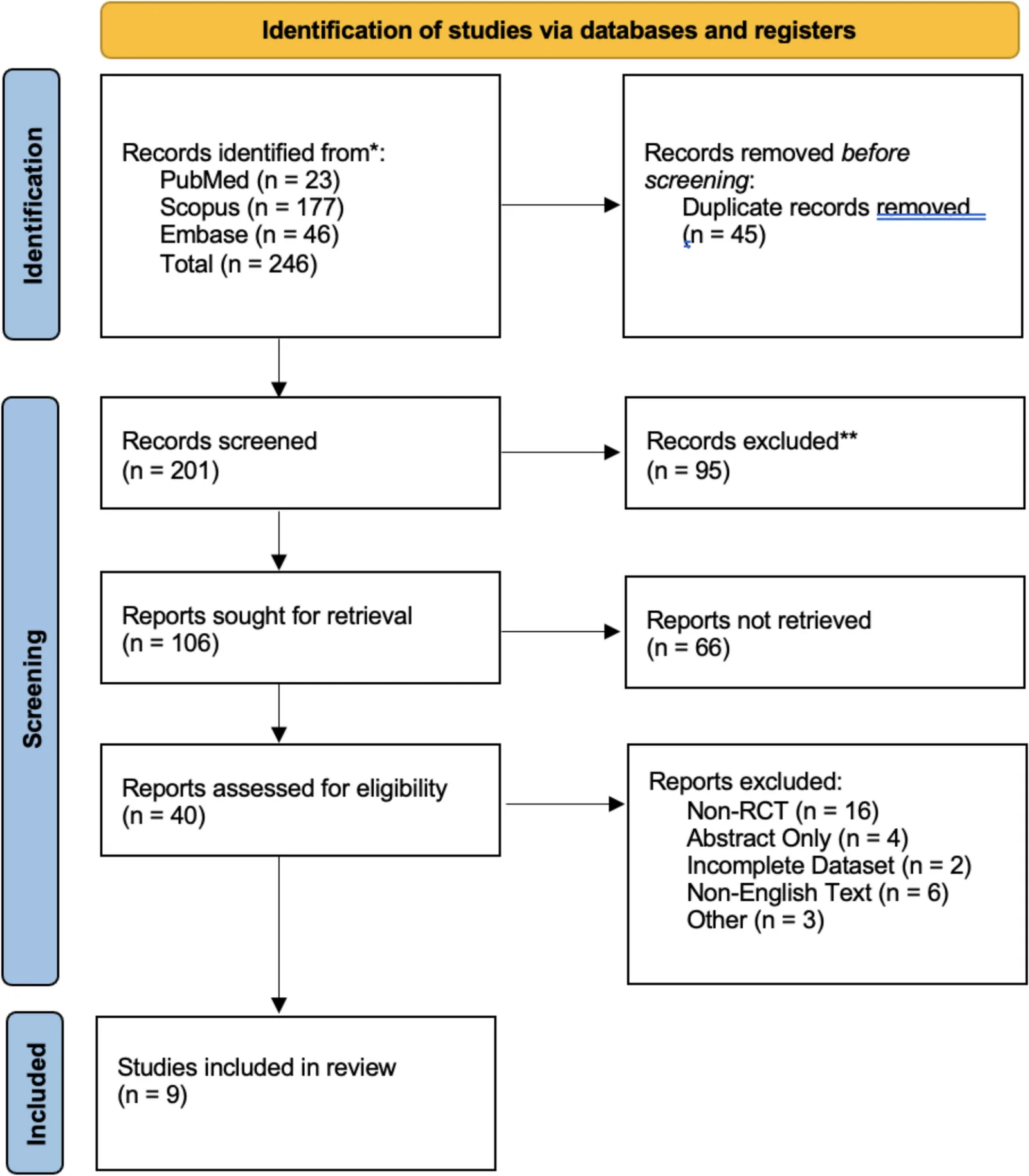
PRISMA flow diagram detailing the process of study identification, screening and inclusion for this meta-analysis

The search of online databases identified a combined total of 246 papers. Following removal of duplicate records, the titles and abstracts of 201 papers were screened for inclusion. Following screening of titles and abstracts, 40 full texts were critically appraised for inclusion. Of these 40 texts, 31 failed to meet the inclusion criteria. Nine RCTs were included in this meta-analysis. The identification, screening and inclusion of relevant papers is summarised in a PRISMA flow diagram (Fig. 1).

### Studies excluded at full text

Several RCTs which evaluated the utility of TXA in TURP failed to meet inclusion were deemed to meet exclusion criteria, the justification for which is detailed herein. Kumsar et al. evaluated IV TXA versus control in a sample of 40 patients undergoing TURP, but did not report parametric statistics and was therefore excluded [19]. Similarly, Rannikko et al. did not report parametric summary statistics, resulting in exclusion [20]. Meng et al. reported significant differences between baseline characteristics of interventional and control cohorts, rendering their results non-comparable [21]. Mirmansouri et al. adopted methodology in which blood transfusion was administered to some patients in the intraoperative period, but these patients were not excluded from perioperative delta haemoglobin outcome reporting, which was deemed to be a significant confounding variable [22].

### Study characteristics

**Fig. 2:**
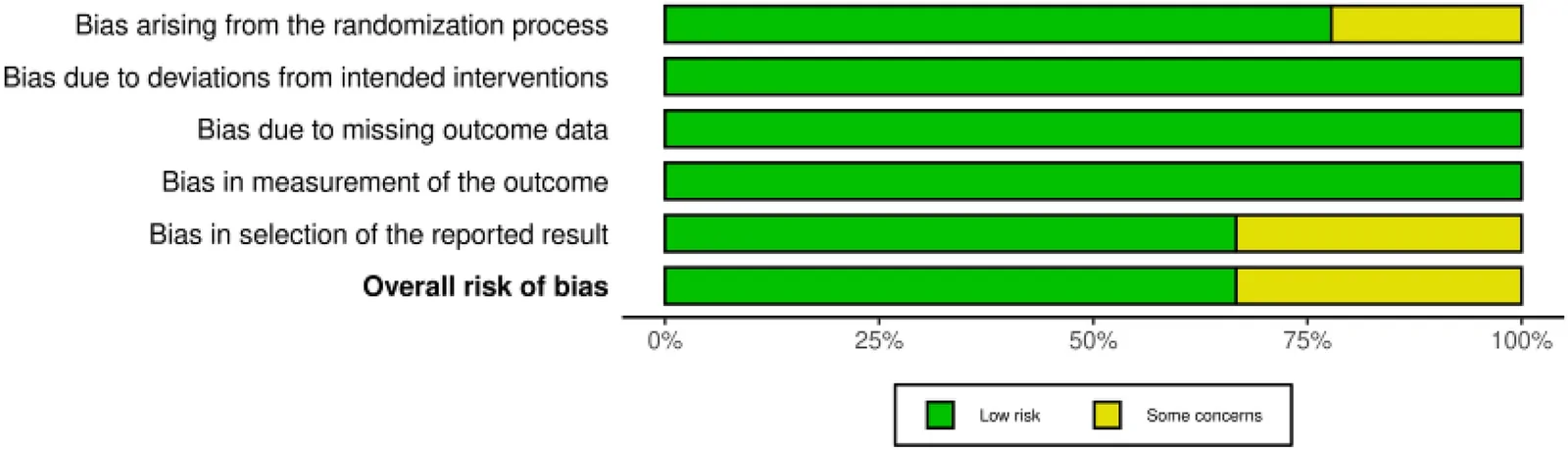
Risk-of-bias summary plot generated from RoB2 assessment

**Fig. 3:**
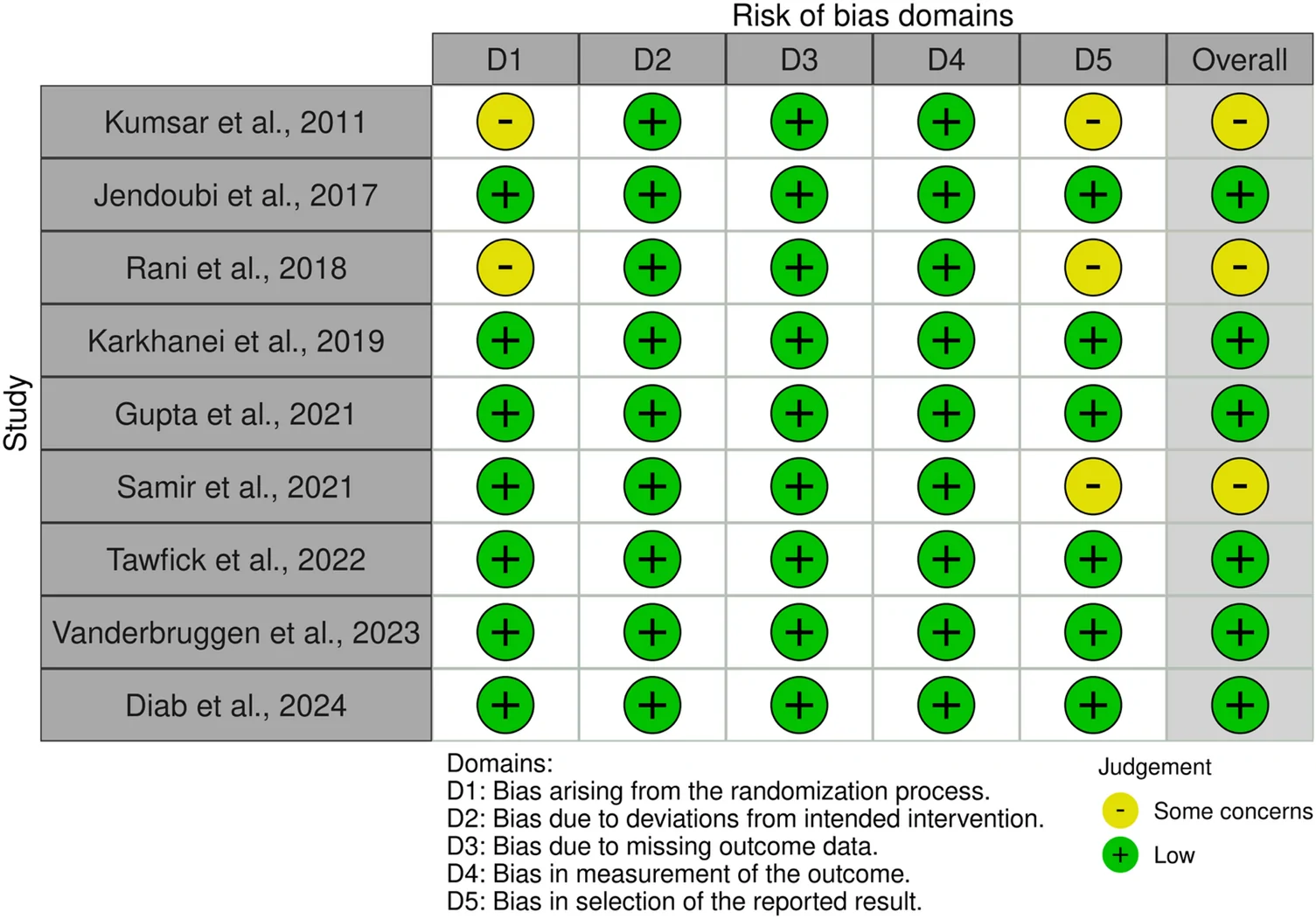
Risk-of-bias traffic light plot generated from RoB2 assessment

Nine RCTs were included in this meta-analysis, published between 2011 and 2024 [11, 12, 19, 23–28] Of the 9 RCTs, 5 were double-blind RCTs [11, 23–26], 1 was a closed-envelope RCT [12], and the remaining 3 were non-blinded RCTs [19, 27, 28]. Seven of the 9 included RCTs were placebo-controlled, whilst the control arm of the remaining 2 studies received TURP without TXA (none) (Table 1). The mode and concentration of TXA administered was heterogeneous across the included studies with TXA administered via IV bolus, IV infusion, and irrigation fluid reported (Fig. 2). All included studies were critically appraised utilising the Cochrane Risk of Bias Assessment Tool (RoB2), demonstrating high evidence quality (Figs. 2, 3).

**Table 1:** Outline of characteristics of included studies

Author	Year	Journal	Country	Design	*n* = int	*n* = ctr	*n* = total
Vanderbruggen et al. [23]	2023	The Prostate	Belgium	Double-blind RCT	31	34	65
Samir et al. [24]	2022	Arab Journal of Urology	Egypt	Double-blind RCT	95	91	186
Tawfick et al. [12]	2022	Arab Journal of Urology	Egypt	Closed-envelope RCT	25	25	50
Diab et al.[11]	2024	Prostatic Disease & Male Voiding	Egypt	Double-blind RCT	30	30	60
Kumsar et al. [19]	2011	Central European Journal of Urology	Turkey	Non-blinded RCT	20	20	40
Jendoubi et al. [25]	2017	Progres en Urologie	Tunisia	Double-blind RCT	30	30	60
Karkhanei et al. [26]	2019	Journal of Clinical Urology	Iran	Double-blind RCT	35	35	70
Rani et al. [27]	2018	International Journal of Scientific Research	India	Non-blinded RCT	30	30	60
Abhimanyu et al. [28]	2021	Urology Annals	India	Non-blinded RCT	35	35	70

*RCT* randomised controlled trial, *n* = sample size, *Int* intervention, *Ctr* Control

### Patient characteristics

Baseline patient characteristics were comparable in all studies, with no statistically significant difference in patient age, prostatic volume, preoperative Hb, ASA grade, and anticoagulant/antiplatelet use nor 5-alpha-reductase inhibitor (5-ARI) use (*p* > 0.05). The mode of TXA administration employed in the respective studies has been outlined (Table 2).

**Table 2:** Outline of mode of administration of TXA in the included studies

Author	Year	Mode of administration	Intervention (TXA)	Control
Vanderbruggen et al. [23]	2023	IV TXA vs placebo	10 mg/kg over 30 min pre-induction followed by 5 mg/kg/hr over 12 h	Equal volume of normal saline 0.9%
Samir et al. [24]	2022	IV TXA vs placebo	50 mg/kg over 20 min pre-induction followed by 5 mg/kg/hr until completion of resection	Equal volume of normal saline 0.9%
Tawfick et al. [12]	2022	Irrigation fluid TXA vs placebo	Intra-op:0.1% TXA: glycine irrigation fluid, postop: clamped catheter 15 min with 500 mg (5 mL) TXA in 100 mL NS	Intra: 5 mL distilled water in 1L glycine irrigation fluid postop: 5 mL distilled water in 100 mL NS
Diab et al. [11]	2024	Intraprostatic TXA injection vs placebo	1 g TXA in 50 mL N/s injected at multiple sites within the prostate	60 mL 0.9% Normal saline injection at multiple sites within the prostate
Kumsar et al. [19]	2011	IV TXA vs none	10 mg/kg TXA over 30 min intraoperatively	None
Jendoubi et al. [25]	2017	IV TXA vs placebo	10 mg/Kg TXA Bolus/30 min at induction, 24 h infusion of 1 mg/kg/hr for 24 h postop	Equal volume of normal saline
Karkhanei et al. [26]	2019	IV TXA vs placebo	500 mg TXA in 500 mL CSL—pre-op bolus 15 mg/kg/20 min, 1 mg/kg/hr infusion until 5 h postop	Equal volume CSL
Rani et al. [27]	2018	Irrigation fluid TXA vs placebo	1 g TXA/ 5L of glycine–irrigation fluid	Equal volume glycine
Abhimanyu et al. [28]	2021	IV TXA + irrigation TXA vs none	Post Induction IV TXA 500 mg, intraoperative irrigation 500mhTXA/3L 0.9% NS (Max 2 g)	None

5 studies administered TXA IV, 2 studies utilised TXA infused irrigation agents, 1 study locally injected TXA into the prostate, and 1 study combined IV TXA and irrigation infused TXA

### Surgical outcomes and complication rates

#### Operative time and endoscopic view quality

**Fig. 4:**
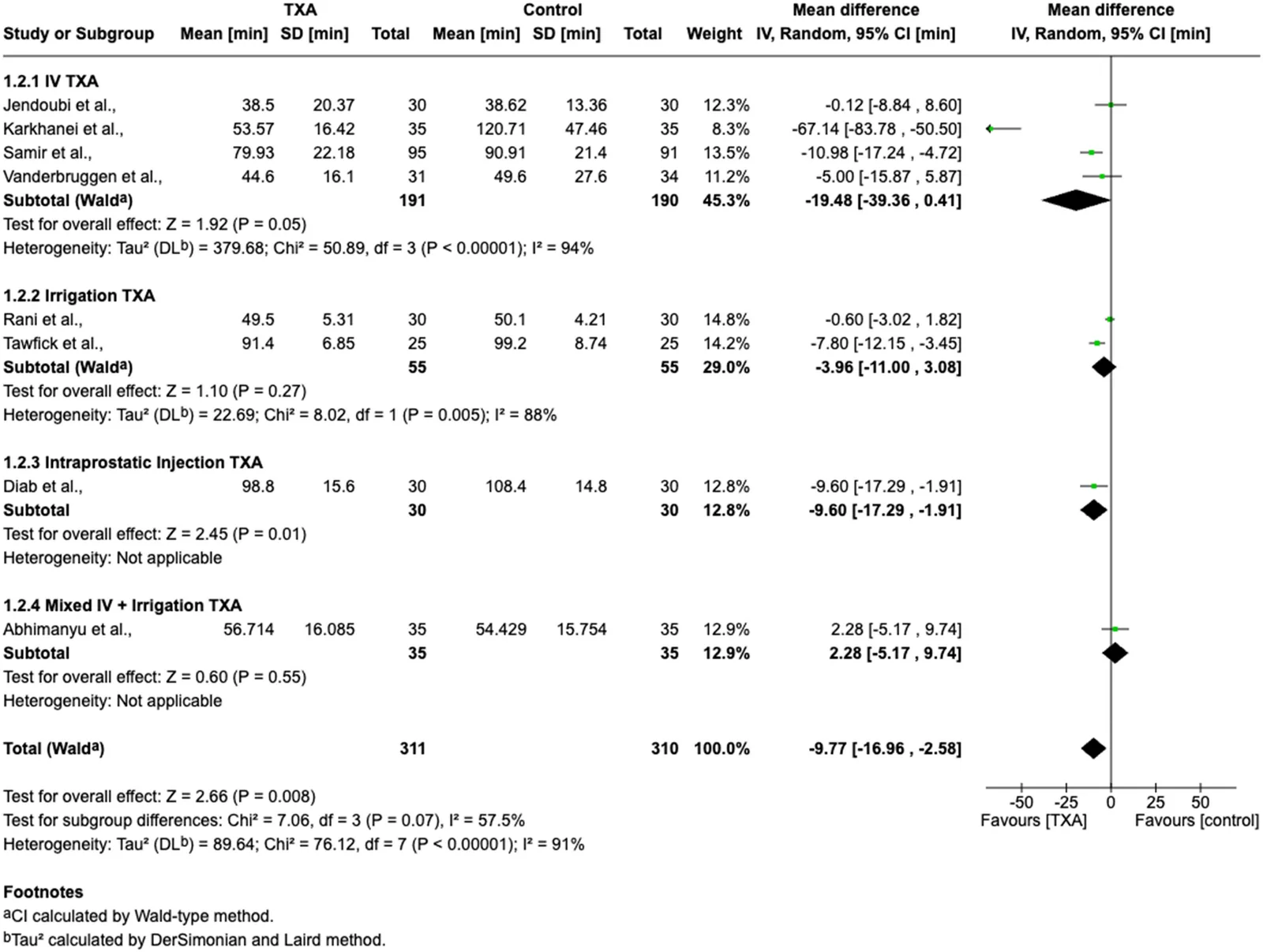
Forest plot demonstrating a statistically significant reduction in operative time in patients treated with TXA vs. Control

Nine RCTs reported operative time, in which 311 patients were treated with TXA and 310 patients served as control. Pooled mean resection time was shorter in TXA-treated patients (63.85 min) compared to control (76.25 min). TXA administration resulted in a mean reduction in operative time of 9.77 min [MD –9.77 min [95%CI –16.97 to –2.58]]. This reduction was statistically significant, *p* = 0.008 (Fig. 4). Subgroup analysis according to the route of TXA administration demonstrated varying effects. Intraprostatic TXA injection resulted in significant reduction in operative time [MD –9.60 min [95%CI –17.29 to –1.91], *p* = 0.01. IV TXA demonstrated a non-significant trend towards shorter operative duration [MD –19.50 min [95%CI –11.00 to 3.08], *p* = 0.27. The combined IV and irrigation TXA approach did not demonstrate a reduction in operative time [MD 2.28 min [95%CI –5.17 to 9.74], *p* = 0.55]. There were no significant differences between subgroups (*χ*^2^ = 7.07, *p* = 0.07), although heterogeneity remained moderate to high (Fig. 4).

Two studies aimed to evaluate the effect of TXA on the quality of endoscopic view (EVQ), which is a likely contributor to the reduced operative time in TXA-treated patients [11, 12]. There was insufficient reporting across included studies to perform meta-analysis on EVQ; however, both Tawfick et al. and Diab et al. demonstrated significantly superior EVQ in TXA-treated patients. Tawfick reported that EVQ was scored as ‘good’ in 56% of TXA-treated resections vs 20% of control, whilst Diab et al. scored EVQ as ‘good’ in 60% of the TXA group vs 26.7% of control (*p* = 0.032 & *p* = 0.03, respectively).

#### Intraoperative blood loss

**Fig. 5:**
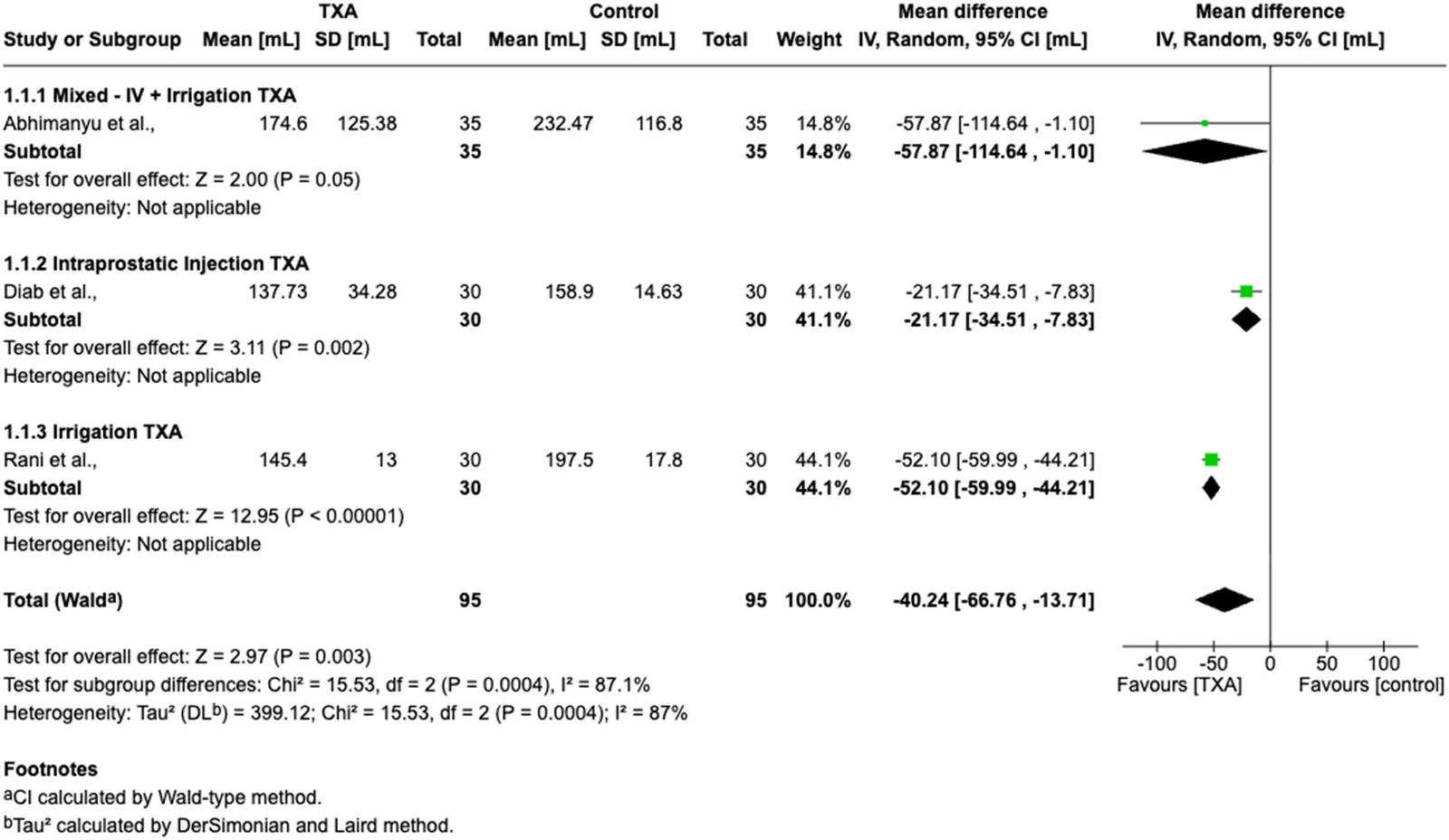
Forest plot demonstrating a statistically significant reduction in intraoperative blood loss in patients treated with TXA vs. Control

Intraoperative blood loss was assessed by analysis of irrigation fluid in three studies. Pooled mean blood loss was 152.58 mL in the TXA arm and 196.29 mL in the control arm. Meta-analysis revealed a mean reduction in intraoperative blood loss of 40.42 mL in patients treated with TXA [MD –40.24 mL [95%CI –66.76 to –13.71]]. This was statistically significant, *p* = 0.003 (Fig. 5). Subgroup analysis was performed in accordance with the mode of TXA administration. All modes of administration demonstrated a reduction in blood loss compared with control. The greatest reduction in intraoperative blood loss was observed with the combined use of both IV and irrigation TXA [MD –57.87 mL [95%CI –114.64 to –1.10], *p* = 0.05. Irrigation TXA also significantly reduced blood loss [MD –52.10 mL [95%CI –59.99 to –44.21], *p* < 0.001], whilst intraprostatic injection of TXA demonstrated a smaller but significant reduction [MD –21.17 mL [95%CI –34.51 to –7.83], *p* = 0.002]. There was statistically significant differences observed between subgroups (*χ*^2^ = 15.53, df = 2, *p* < 0.001) (Fig. 5). The number of studies available per subgroup was small, and these results should be interpreted as exploratory.

**Fig. 6:**
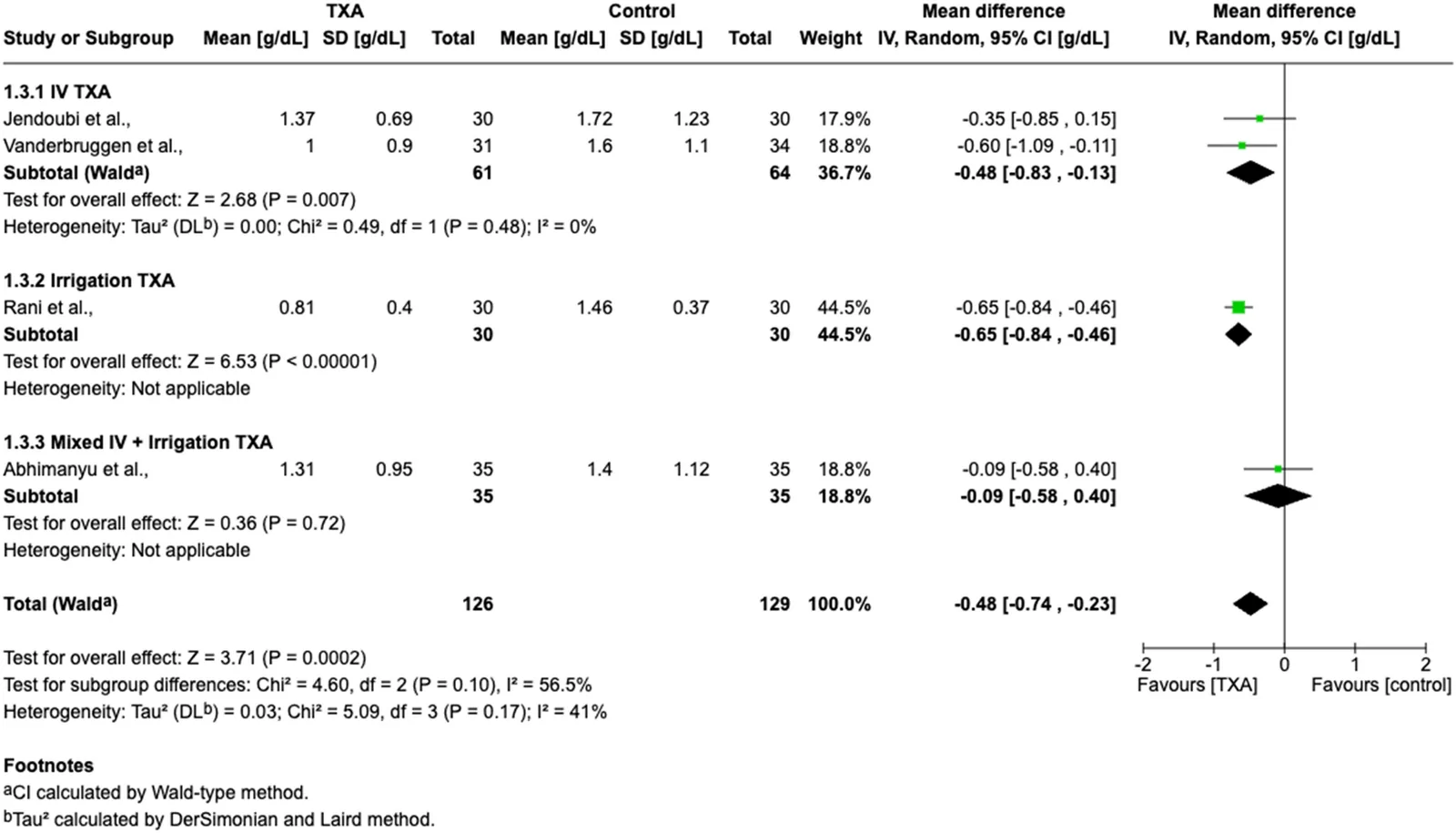
Forest plot demonstrating a highly significant reduction perioperative blood loss as demonstrated in by mean difference between preoperative Hb and Hb values on the first postoperative day. There was a statistically significant reduction in Hb drop associated with intraoperative TXA

Perioperative blood loss was further evaluated by 4 of the included studies by calculating the difference in patient Hb values taken preoperatively and on the first postoperative day (*Δ*Hb). *Δ*Hb in TXA-treated patients was significantly less than the control [MD –0.55 g/dL [95%CI –0.71 to –0.39], *p* < 0.00001] (Fig. 6). Similarly, subgroup analysis was performed based on the route of TXA administration for the *Δ*Hb variable. Irrigation TXA demonstrated the largest reduction in *Δ*Hb [MD –0.65 g/dL [95%CI –0.84 to –0.46], *p* < 0.001]. IV TXA also demonstrated a significant reduction [MD –0.48 g/dL [95%CI –0.83 to –0.13, *p* = 0.007]. Combined IV and irrigation TXA did not demonstrate a significant effect [MD –0.09 g/dL [95%CI –0.58 to 0.40], *p* = 0.72]. There were no significant differences observed between subgroups (*χ*^2^ = 4.60, *p* = 0.10) (Fig. 6).

Subgroup analyses for blood loss outcomes were also performed comparing monopolar and bipolar TURP studies. For intraoperative blood loss, both bipolar and monopolar TURP demonstrated significant reduction in blood loss with TXA administration (Supplementary Fig. 1). Similarly, when analysing *Δ*Hb, both bipolar and monopolar TURP demonstrated lower mean *Δ*Hb values, albeit non-significant, with intraoperative TXA (Supplementary Fig. 2).

#### Blood transfusion

**Fig. 7:**
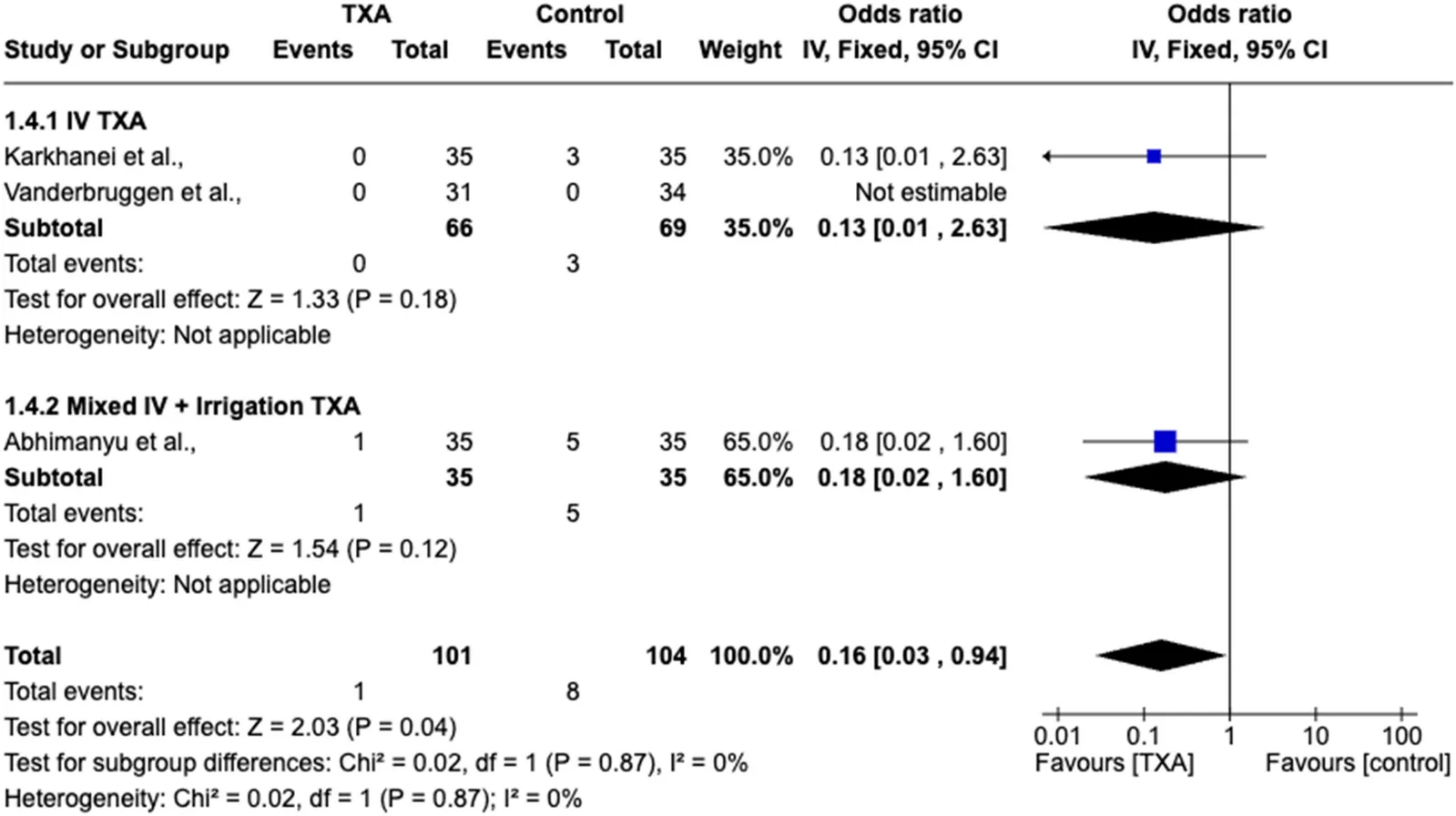
Forest plot demonstrating the incidence of blood transfusion requirement in patients who underwent TURP treated with TXA vs. Control. There was a significant reduction in blood transfusion requirement associated with intraoperative TXA

Incidence of blood transfusion post-TURP was recorded in three of the included RCTs, in which 205 patients underwent TURP (101:104, TXA:Control). The pooled analysis demonstrated that 0.99% (*n* = 1/101) of TXA-treated patients required blood transfusion as opposed to 7.69% (*n* = 8/104) of patients in the control arm. Meta-analysis demonstrated risk of blood transfusion requirement to be significantly less in TXA [OR 0.16 [95%CI 0.03–0.93]], this relationship reached statistical significance at *p* = 0.04 (Fig. 7). Subgroup analysis according to the route of TXA administration demonstrated similar trends. IV TXA demonstrated a non-significant trend towards reduced blood transfusion requirement [OR 0.56 [95%CI 0.16–1.93], *p* = 0.36] as did combined IV and irrigation TXA administration [OR 0.18 [95%CI 0.02–1.60], *p* = 0.12]. The test for subgroup differences was non-significant (*χ*^2^ = 0.81, *p* = 0.37) (Fig. 7).

#### Deep vein thrombosis and pulmonary emboli

**Fig. 8:**
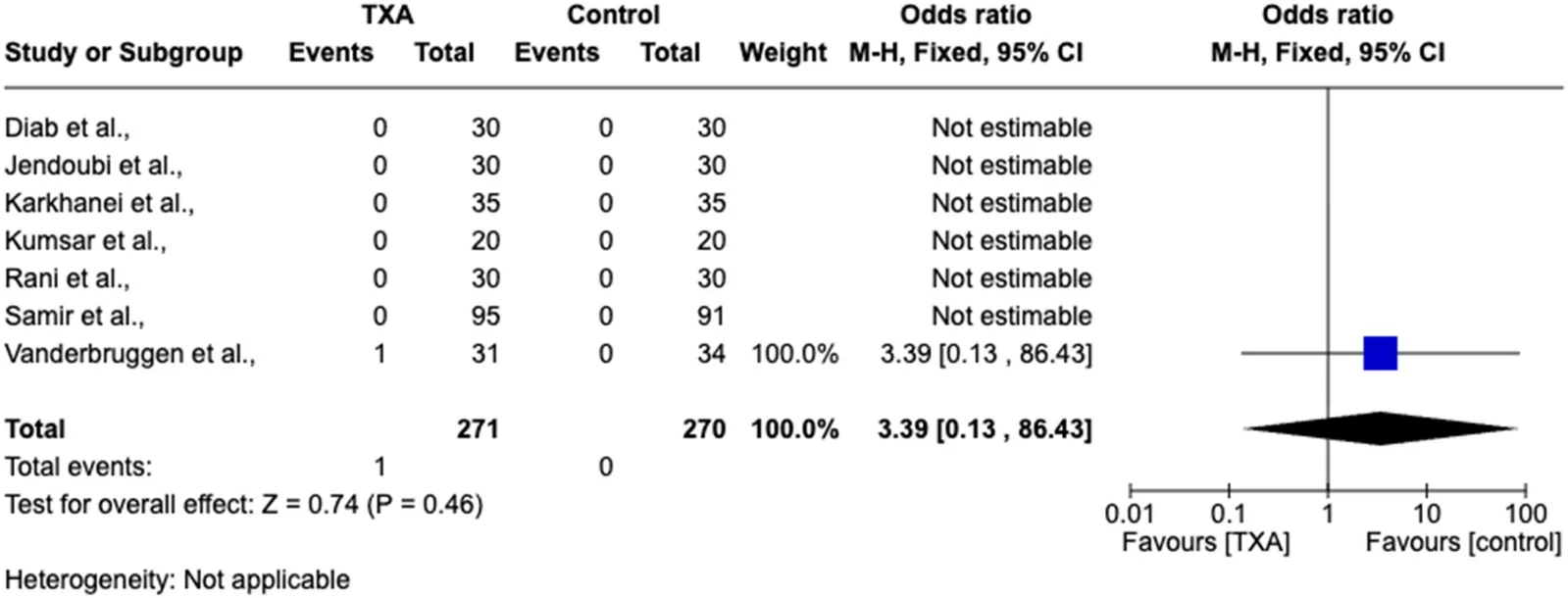
Forest plot demonstrating the incidence of DVT amongst patients undergoing TURP treated with TXA vs. Control. There was no statistically significant increase in risk of DVT imposed on TXA-treated patients (*p* > 0.05)

Seven of the included studies reported the incidence of deep venous thrombosis (DVT) across TXA and control study cohorts, in which 541 patients underwent TURP (271:270, TXA:Control). There was one DVT reported in the pooled intervention arm (*n* = 1/271, 0.37%), with no DVT reported in the control arm (*n* = 0/270). Meta-analysis demonstrated no statistically significant increase in DVT risk imposed on patients treated with TXA [OR 3.39 [95%CI 0.13–86.43]], *p* = 0.46. (Fig. 8).

Pulmonary embolism incidence was specifically reported in four of the included studies [11, 19, 24, 25], which reported data representative of 346 patients who underwent TURP (175:171, TXA:Control). No pulmonary embolism was observed within either arm of the pooled cohort. Meta-analysis was not-performed as all included trials reported double-zero events for this variable.

#### Secondary outcomes

The length of time required before continuous bladder irrigation (CBI) could be discontinued was assessed in 3/9 included studies, in which 311 pooled patients underwent TURP (156:155, TXA:Control). TXA-treated patients demonstrated shorter time to CBI discontinuation 22.46 h in TXA vs 27.54 h in control [MD –4.13 h [95%CI –10.18 to 1.92]]; this trend was not statistically significant; however, *p* = 0.18 (Supplementary Fig 3). Two of the included studies reported a significant decrease in duration of CBI [23, 24]. One study reported longer duration of CBI in TXA-treated patients [25]. The heterogeneity in TXA route of administration and dosages in combination with the variable effect direction renders this analysis underpowered.

Similarly, 3/9 included studies reported time to decatheterisation (156:155, TXA:Control). Mean time to decatheterisation was shorter in TXA-treated patients, 40.24 h in TXA vs 44.19 h in control [MD –2.41 h [95%CI –8.03 to 3.22]]; this did not reach statistical significance, *p* = 0.40 (Supplementary Material Fig. 4). Similarly, the direction of effect was varied in this outcome, rendering this outcome underpowered for interpretation.

Time to discharge was reported by 4/9 included studies representative of *n* = 371 patients who underwent TURP, (186:185, TXA:Control). Mean time to discharge was 56.13 h in TXA-treated patients and 60.25 h in the control group. Mean time to discharge was 1.78 h shorter in TXA-treated patients [MD –1.78 [95%CI –4.71 to 1.15]]; this finding did not reach statistical significance, *p* = 0.23 (Supplementary Material Fig. 5). Although the direction of effect was towards reduction in three of four included studies, this outcome remains underpowered for adequate assessment at present.

The incidence of clot retention is of interest given the theoretical increased risk of clot formation with TXA administration. This outcome was reported in four of the included studies, which reported data on *n* = 235 patients undergoing TURP (126:129, TXA:Control) [11, 23, 26, 27]. Clot retention occurred in *n* = 2/126 (1.6%) of the patients treated with TXA, compared to *n* = 12/129 (9.3%) of the control cohort. Random-effects meta-analysis demonstrated a significant reduction in clot retention associated with TXA administration [OR 0.16 [95%CI 0.04–0.68], *p* = 0.01] (Supplementary Material Fig. 4).

## Discussion

This systematic review and meta-analysis has demonstrated TXA to be efficacious in reducing perioperative bleeding in TURP. The pooled data indicated a statistically significant mean reduction in intraoperative blood loss (–40 mL) and *Δ*Hb on the first postoperative day (–0.5 g/dL) in TXA-treated patients. The 40 mL mean reduction in intraoperative blood loss alone is of uncertain clinical relevance. Typical TURP-associated blood loss is reported at ≈200 mL [29]. The pooled mean blood loss in our control group was consistent with this value at 198.2 mL. Intraoperative blood loss exceeding 500 mL is broadly cited as the threshold for clinical significance within surgical literature, with previous National Institute for Clinical Excellence (NICE) guidelines recommending routine TXA utilisation in surgeries in which *a* > 500 mL blood loss is expected [30]. However, given the typical age and comorbidity profile of patients undergoing TURP, more conservative blood loss targets represent a worthy risk-reduction pursuit. A recent meta-analysis reported a similarly modest mean reduction in intraoperative blood loss (–38 mL) amongst general surgical patients receiving TXA [31]. Critically, this resulted in a significant decrease in risk of blood transfusion requirement [OR 0.75 [95%CI 0.60–0.94), *p* = 0.01]. Similarly, this article demonstrates that modest reductions in intraoperative blood loss (≈40 mL) during TURP is associated with an associated significant reduction in blood transfusion requirement (OR 0.16, *p* = 0.04). As such, the clinical relevance of TXA as a pharmacological adjunct in TURP becomes more certain. Notably, the updated 2026 NICE guidelines coincide with this data, now recommending the utilisation of TXA in all adult patients undergoing surgery in which there is “*any risk of bleeding and the procedure will break the skin or mucous membranes”*. The risks associated with blood transfusion are multiple and have been well documented in the literature [32]. Limiting patient exposure to potential blood transfusion-related morbidity should be a primary consideration for those considering TXA use in TURP.

Furthermore, this meta-analysis demonstrates TXA to be efficacious in reducing operative time in TURP (*p* = 0.008). Our group hypothesise that improved endoscopic visualisation owed to decreased intraoperative bleeding is a likely contributing factor to this finding. The effect of TXA on endoscopic view quality (EVQ) was evaluated in two of the included RCTs, but did not meet the threshold for meta-analysis [11, 12]. Both Tawfick and Diab assessed EVQ through surgeon rated endoscopic visibility, as rated on a scale of 1–3. Although such rating of endoscopic view is inherently subjective, it should be noted that both authors demonstrated EVQ to be significantly superior in TXA-treated patients (*p* < 0.05). This finding is somewhat limited however, in the case of Tawfick, whose study design utilised a closed-envelope randomisation method, rendering surgeons unblinded at the time of scoring. Diab however, utilised a double-blind study design. Further evaluation of this finding should ensure that scoring surgeons are blinded at the time of scoring to limit potential bias.

The primary concern limiting perioperative utilisation of TXA has been centred around potential increased risk of thromboembolism (TE). Numerous studies have demonstrated a negligible increase in TE when TXA is utilised intraoperatively across a plethora of surgical sub-specialities [16, 33]. This study presents a meta-analysis of high-quality data specific to TURP and demonstrates TXA to incur a negligible increase in thromboembolic risk. Although our study presents a comparatively smaller sample size, our results remain comparable to larger meta-analysis of RCTs evaluating routine use of TXA in orthopaedic lower limb surgery (*n* = 15,659) which too demonstrated no significant increase in VTE occurrence (2.8% TXA vs 2.4% Control, [OR 1.10 [95%CI 0.92 to –1.32], *p* = 0.291] [34]. Furthermore, patients post-TURP are likely to return to baseline mobility earlier than their orthopaedic counterparts in the postoperative period, rendering them less vulnerable to VTE.

This systematic review was subject to a number of limitations. The mode of administration of TXA was heterogeneous amongst the included studies, with IV being the preferred route in the majority (*n* = 5) of RCTs. Alternative routes of administration, such as irrigation fluid and intraprostatic injection, were not reported in sufficient number to permit subgroup analysis. Urine has naturally occurring antifibrinolytic properties. Hence, inclusion of a potent inhibitor of the fibrinolysis pathway such as TXA to irrigation solution presents a sensical solution to overcoming this physiological barrier to haemostasis in TURP. However, IV administration remains the modality of TXA with the most robust evidence base to date. To objectively evaluate the optimal mode and dose of TXA for TURP, further prospective, randomised studies are required. The heterogeneity of blinding strategies employed in the included studies is a further limitation. Although all studies were found to be acceptable upon RoB analysis, further studies should strive for double-blind placebo-controlled study design. This is especially pertinent if EVQ is to be assessed with subjective scoring mechanisms was employed by both Tawfick and Diab [11, 12].

This article demonstrates TXA to be effective in reducing intraoperative blood loss, operative time and postoperative reduction in haemoglobin in TURP. Consequently, patients who receive intraoperative TXA are at less risk of requiring blood transfusion. Intraoperative TXA administration in TURP is safe and is not associated with any significant increase in thromboembolic events.

## Supplementary Information

Below is the link to the electronic supplementary material.

Supplementary file1 (DOCX 544 KB)

## Data Availability

All data included in this analysis is publicly available through peer-reviewed publication. New data generated through meta-analysis is reported within the text, figures and supplementary materials.
