# Supplementary material for: The role of intraoperative tranexamic acid in transurethral resection of the prostate: a systematic review and meta-analysis of randomised-controlled trials
Source: Int Urol Nephrol. 2026 May 7;58(10):4003–14. doi: 10.1007/s11255-026-05180-7 (PMC13585812; doi:10.1007/s11255-026-05180-7)
Supplement: Supplementary file 1 [file 11255_2026_5180_MOESM1_ESM.docx]

**Supplementary Figure 1.** *Forest plot demonstrating subgroup comparison of intraoperative blood loss between monopolar, bipolar and non-specified TURP patients.*

**Supplementary Figure 2. *Forest plot demonstrating subgroup comparison of delta Hb between monopolar, bipolar and non-specified TURP patients.***

**Supplementary Figure 3.** *Forest plot demonstrating a non-significant reduction in duration of continuous bladder irrigation in patients who underwent TURP treated with TXA vs. Control.*

**Supplementary Figure 4.** *Forest plot demonstrating a non-significant reduction in time to decatheterisation in TURP patients treated with TXA vs. Control.*

**Supplementary Figure 5.** *Forest plot demonstrating a non-significant reduction in time to discharge/ length of stay, in TURP patients treated with TXA vs. Control.*

**Supplementary Figure 6.** *Forest plot demonstrating a significant reduction in the incidence of postoperative clot retention among TXA treated patients undergoing TURP compared to control.*
